# An Open-Source Automated Pipeline for Quantitative Morphological Analysis of 3D-Bioprinted Cancer Cell Spheroids

**DOI:** 10.3390/mps9010021

**Published:** 2026-02-02

**Authors:** Pius N. Amartey, Jocelyn S. Kim, Yetunde I. Kayode, Glenn E. Simmons

**Affiliations:** Department of Biomedical Sciences, College of Veterinary Medicine, Cornell University, Ithaca, NY 14850, USA; pna27@cornell.edu (P.N.A.); jsk379@cornell.edu (J.S.K.); yik3@cornell.edu (Y.I.K.)

**Keywords:** 3D culture, bioprinting, tumor metastasis, tumor microenvironment, open source, breast cancer

## Abstract

Three-dimensional (3D) culture systems that recapitulate the tumor microenvironment are essential for studying cancer cell behavior, drug response, and cell–matrix interactions. Here, we present a detailed protocol for generating 3D spheroid cultures from murine breast cancer cells using methacrylated gelatin (GelMA)-based bioink and a CELLINK BioX bioprinter. This method enables precise deposition of spheroid-laden GelMA droplets into low-attachment plates, facilitating high-throughput and reproducible 3D culture formation. The protocol includes steps for spheroid formation, GelMA preparation, bioprinting, and post-printing analysis using a customized CellProfiler pipeline. The analysis pipeline takes advantage of the functionality of CellProfiler and ImageJ software (version 2.14.0) packages to create a versatile and accessible analysis tool. This approach provides a robust and adaptable platform for in vitro cancer research, including studies of metastasis, drug resistance, cancer cell lipid metabolism, and TME-associated hypoxia.

## 1. Introduction

For decades, two-dimensional (2D) cell culture models have served as a cornerstone of cancer research due to their low cost, ease of use, and high-throughput compatibility [1,2,3]. These models have played a pivotal role in helping elucidate cellular mechanisms, signaling pathways, and drug responses. However, their inherent lack of biological complexity often limits their translatability to in vivo systems and clinical applications [4,5,6].

In recent years, three-dimensional (3D) in vitro models, such as spheroids and organoids, have gained prominence as tools that more accurately recapitulate the architecture, heterogeneity, and microenvironment of tumors [7,8,9,10,11]. One of the primary physiological advantages of spheroids is their ability to mimic the spatial organization of cells found in solid tumors, including the formation of nutrient and oxygen gradients, resulting in hypoxic cores and proliferation-restricted zones, conditions that closely resemble in vivo tumor physiology [10,12,13]. In this context, gelatin methacryloyl (GelMA) hydrogels have emerged as highly adaptable biomaterials for 3D cell culture [14,15,16]. GelMA offers tunable mechanical properties, high biocompatibility, and the capacity for photopolymerization, enabling precise spatial control during printing or casting. These properties make GelMA particularly well suited for forming and supporting multicellular spheroids over extended culture periods of up to two weeks with minimal user intervention. When used in 3D co-culture models, GelMA allows for robust incorporation of multiple cell types, facilitating more physiologically relevant studies of cell–cell and cell–matrix interactions [9,10,13,16,17]. Furthermore, bioprinting technologies, such as those offered by CELLINK’s BioX platform, enable the structured deposition of cell-laden GelMA bioinks into defined architecture [18,19]. This use of biofabrication significantly improves spatial reproducibility, scalability, and compatibility with high-content imaging, advantages that are difficult to achieve using traditional drop-seeding or scaffold-free approaches [7,20,21,22].

The goal of this study was to develop and validate a reproducible and rapid image analysis framework for scalable 3D tumoroid co-cultures using GelMA matrices. Specifically, we aimed to test our ability to: (1) generate spheroids using bioprintable GelMA-based bioinks containing cancer cells, (2) maintain stable cultures for long-term observation and longitudinal imaging, and (3) investigate responses to exogenous agents (i.e., fatty acids) within a dynamically interacting tumor microenvironment using our analysis pipeline. Our long-term objective is to eventually incorporate this procedure into a broader drug/therapeutic testing workflow compatible with advanced bioimaging and high-throughput data processing. As a proof of concept, we utilized GelMA-embedded murine breast cancer (PYMT) spheroids to model tumor morphology during epithelial–mesenchymal transition (EMT), with a particular focus on the influence of monounsaturated fatty acids on tumor morphology.

**Figure 1:**
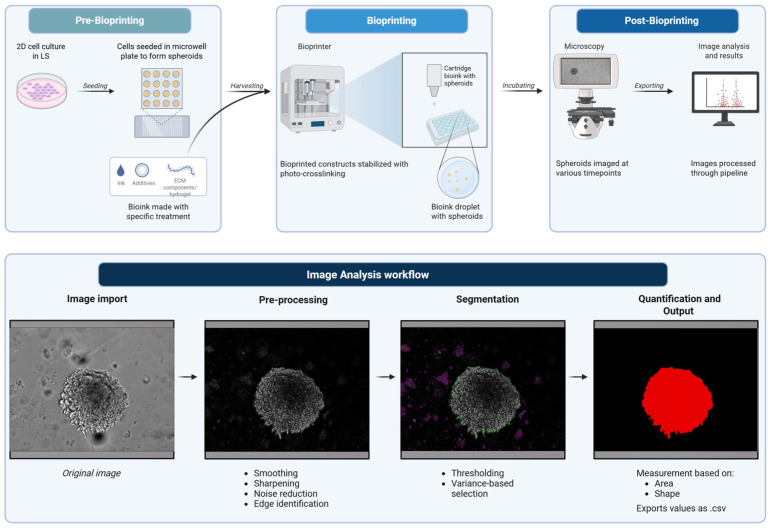
Schematic of bioprinting and image analysis pipeline. Created in BioRender. Amartey, P. (2026) https://BioRender.com/zz2g883. (accessed on 17 January 2026).

Our work here demonstrates that the GelMA-based cell culture platform can provide a highly controllable and scalable approach for generating physiologically relevant 3D tumor models. The integration of advanced bioprinting, prolonged culturing, and automated image analysis is suited for high-throughput drug screening, investigation of tumor–stroma interactions, and personalized therapeutic modeling. Thus, by bridging critical gaps between traditional 2D culture and in vivo systems, the combination of this 3D model and analysis pipeline (Figure 1) contributes to a robust experimental framework for the next generation of cancer research.

## 2. Experimental Design

### Materials

- Sterile PhotoGel^®^ 50% DS (500 mg, CELLINK, Gothenburg, Sweden; VL3500000502).
- Lithium phenyl-2,4,6-trimethylbenzoylphosphinate photoinitiator (LAP) (CELLINK, Gothenburg, Sweden; 5269-500MG).
- The PyMT cell lines, including the PyMT epithelial cell line (pB2) and the SnailHi PyMT quasi-mesenchymal (qM) cell line (pB3), are established murine breast cancer cell lines. The pB2 and pB3 cell lines were kindly gifted to us by Dr. Anushka Dongre. Their establishment was previously described in the published paper “Epithelial-to-mesenchymal transition contributes to immunosuppression in breast carcinomas” by Dongre et al. [23]. Cells were cultured in DMEM/F12 medium containing 5% adult bovine serum (ABS) supplemented with 1× penicillin–streptomycin and 1× non-essential amino acids throughout the study.
- AggreWell™ 800 plate (STEMCELL Technologies, Vancouver, BC, Canada; Cat # 34815).
- 10 mM OA–BSA (oleic acid–bovine serum albumin conjugate) (Sigma-Aldrich, St. Louis, MO, USA; Cat# O3008-5ML).
- Bovine serum albumin (BSA) (ThermoFisher, Waltham, MA, USA; Cat # J64944-22).
- Complete PyMT media (Table 1 for composition).
- 0.25% Trypsin–EDTA (Corning, Corning, NY, USA; Cat# 45000-664).
- 100 mm cell culture-treated dishes.
- 15 mL Falcon tubes.
- Amber cartridge, 3 cc (CELLINK, Gothenburg, Sweden; Cat# CSO010311502).
- Steriflip Millipore 0.22 µM vacuum filter (Sigma-Aldrich, St. Louis, MO, USA, Cat# SCGP00525).
- CELLINK BioX Bioprinter (software version 1.8.2).
- Ultralow-attachment 24-well plates (Corning Cat# 3473).
- CellProfiler software (Cambridge, MA, USA version 4.2.7).

## 3. Procedure

### 3.1. Cell Culture Preparation

#### 3.1.1. Thawing and Expansion

1. Thaw mouse mammary tumor cell lines pB2 and pB3 from frozen stocks.
2. Plate cells in culture medium.
3. Incubate all cultures in a humidified incubator at 37 °C with 5% CO_2_.
4. Passage pB2s 1:10 and pB3s 1:5 every 2–3 days at least three times before experimental use. Cells under passage 15 were used for bioprinting.

#### 3.1.2. Subculturing and Phenotypic Validation

1. Detach cells from culture plates using 0.25% trypsin–EDTA.
2. Re-seed and culture cells for an additional 3 days.
3. Confirm pB2 (epithelial phenotype) and pB3 (mesenchymal phenotype) characteristics by phenotypic marker expression.

#### 3.1.3. Serum Starvation

1. To limit endogenous unsaturated fatty acid availability, replace culture medium with 1% ABS media for one full passage.
2. Maintain separate groups in 1% and 5% serum for comparative analysis of serum-derived lipid effects.

#### 3.1.4. Seeding for 3D Culture

1. Harvest pB3 cells with 0.25% trypsin–EDTA.
2. Count cells to obtain desired cell concentration.
3. Seed 750,000 pB3 tumor cells per well into AggreWell™ 800 plates pre-filled with low serum (1% ABS) PyMT medium.
4. Incubate plates at 37 °C, 5% CO_2_ for 72 h.
5. Monitor spheroid formation using light microscopy.
6. Extend incubation if spheroids are not fully formed (taking on a compact ball appearance) after 72 h.

#### 3.1.5. Harvesting Spheroids

1. Aspirate 50% of the medium from each well of the AggreWell™ 800 plate.
2. Dislodge spheroids using a 1 mL pipette tip with a widened bore (approximately 2–3 mm in diameter) to minimize spheroid damage.
3. Collect spheroid suspension into a 15 mL conical tube, allow spheroids to sediment, then aspirate the supernatant.

#### 3.1.6. Preparation of GelMA and Media Solutions

##### Phase I

1. Turn on the incubator to 50 °C.
2. Retrieve a vial of PhotoGel 50% DS from –20 °C storage and allow it to warm up to 27 °C in a water bath.
3. For 0.10% LAP concentration, obtain an equal mass of LAP for volume of media. For example, if using 12 mL, weigh out 12 mg of LAP (note: from this stage, shield all tubes from direct light).
4. Mix LAP with the media in a canonical tube, wrap with foil, and incubate at 50 °C for about 15 min (or until solution is clear).
5. Sterilize the LAP–medium mixture using the Steriflip Millipore 0.22 µM vacuum filter.
6. Keep the LAP–medium mixture at 50 °C in the incubator.

##### Phase II

7. Retrieve vial of PhotoGel^®^ 50% DS and LAP–medium mix.
8. For 5% GelMA bioink (*w*/*v*) concentration, pipette 10 mL of LAP–medium mix into the vial of PhotoGel^®^ 50% DS.
9. Wrap the tip of the vial with parafilm to prevent the cap from bursting under pressure.
10. Incubate at 50 °C on a rocker for 30 min.
11. Remove parafilm slowly following the 30 min incubation to prevent any accidents.
12. Allow GelMA to cool to 37 °C before use or store at 4 °C.

#### 3.1.7. Culture Medium Preparation

1. Equilibrate 24 mL of complete PyMT culture medium to 37 °C (1 mL for each well planned).
2. Prepare media containing either BSA or OA–BSA as follows:
  a. Tube 1: 90 µL of 10 mM OA–BSA in 5.91 mL medium (final 150 µM OA–BSA).
  b. Tube 2: 45 µL of 10 mM OA–BSA + 45 µL of 10 mM BSA in 5.91 mL medium (final 75 µM OA–BSA + 75 µM BSA).
  c. Tube 3: 30 µL of 10 mM OA–BSA + 60 µL of 10 mM BSA in 5.91 mL medium (final 50 µM OA–BSA + 100 µM BSA).
  d. Tube 4: 90 µL of 10 mM BSA in 5.91 mL medium (final 150 µM BSA).
  e. (Optional) Filter solutions if necessary to remove particulates.

#### 3.1.8. Bioprinter Calibration and Droplet Printing

1. Power on the CELLINK BioX bioprinter, select the droplet print, and then select 24-well plate format.
2. Set printhead temperature control to 27 °C, print bed to 20 °C, pressure to 30 kPa, extrusion time of 0.7 s, and 405 nm cross-link exposure time to 5 s, optimized from previous methods [24].

#### 3.1.9. GelMA–Lipid Formulations

1. Thaw 5% GelMA solution from −20 °C if not already freshly made.
2. Divide 1.97 mL aliquots of GelMA into four foil-wrapped tubes and add as follows:
  a. Tube 1: 30 µL of 10 mM OA–BSA.
  b. Tube 2: 15 µL of 10 mM OA–BSA + 15 µL of 10 mM BSA.
  c. Tube 3: 10 µL of 10 mM OA–BSA + 20 µL of 10 mM BSA.
  d. Tube 4: 30 µL of 10 mM BSA.

#### 3.1.10. Spheroid–GelMA Mixing

1. Combine spheroids with GelMA by adding 2 mL of the appropriate GelMA solution to each tube containing spheroids.
2. Gently invert tubes to ensure homogeneous spheroid suspension within the GelMA.
3. Load spheroid–GelMA mixtures using a wide-bore (2–3 mm in diameter) 1 mL pipette tip into pre-labeled amber cartridges.
4. Insert the cartridge piston, expel any air bubbles, and secure cartridges with caps on both ends (as per manufacturer instructions).
5. If GelMA is too fluid (air bubbles moving within cartridge), chill at 4 °C for 2 min. If GelMA is too stiff (not able to flow with gentle pressure), incubate cartridges at 37 °C for 2–4 min.
6. Dispense droplets using bioprinter into low-attachment 24-well plate using 30 kPa of pressure, an extrusion time of 1.5–4 s, and cartridge temperature of 27 °C. Droplets should be centered within the well. (Any problems with gel consistency or flow see Table 2 for potential fixes).
7. Optimize extrusion time for droplet formation as needed.
8. Stabilize GelMA constructs via cross-linking with a 405 nm LED light source for 5 s at a distance of 3 cm to activate the LAP photoinitiator for each droplet that has been dispensed.

#### 3.1.11. Media Addition and Incubation

1. Add 1 mL of media from the tubes containing either BSA or OA–BSA at different concentrations (from Section 3.1.4) to the corresponding lipid-treated droplet.
2. Incubate the plate in a humidified incubator at 37 °C with 5% CO_2_. This is day 0.
3. Image spheroids every 2–3 days to observe and document changes in morphology.

### 3.2. Post-Printing Analysis

#### 3.2.1. Microscopy

**Figure 2:**
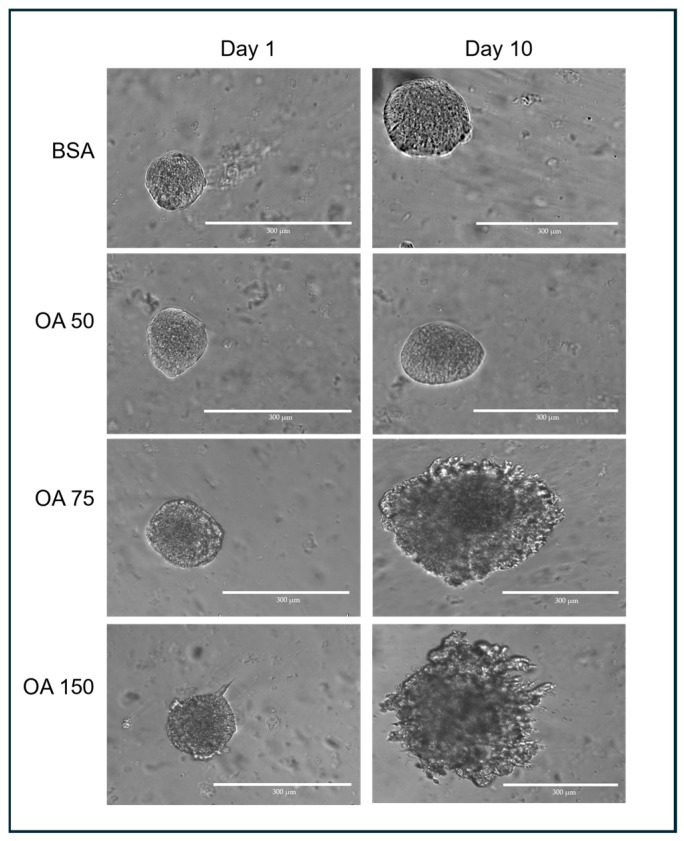
Representative spheroids following treatment with different concentrations of OA–BSA for 10 days.

Transverse images of 3D spheroids were captured using an EVOS M5000 microscope (ThermoFisher, Waltham, MA, USA) at 10× magnification (Figure 2). When imaging the spheroids, the ideal target spheroids are trackable and isolated in the region of interest at 4× magnification. The z-distance from the bottom of the culture plate was first determined, and spheroids that were at least 500 µm away or more were captured. This was to ensure that spheroids captured were not those growing on the surface of the plate outside the GelMA. The appropriate focal plane (z-distance) was assessed manually for each spheroid and exported in TIFF format.

#### 3.2.2. Image Analysis

Post-printing analysis of spheroids was conducted using CellProfiler version 4.2.7. CellProfiler is an open-source cell image analysis software that allows high-throughput quantitative analysis of images [25]. CellProfiler performs quantitative image analysis by helping the user construct pipelines that consist of assembling any number of modules to perform specific tasks to measure unique features in the images of choice. In our spheroid analysis pipeline, the 4 input modules (Images, Metadata, NamesAndTypes, Groups) are supplemented with 7 other modules (ColorToGray, RunImageJMacro, GaussianFilter, EnhanceEdges, IdentifyPrimaryObjects, MeasureObjectSizeShape, ExportToSpreadsheet). The pipeline modifies the input data into a mask that allows for the border of the spheroids to be easily identifiable and measures the physical characteristics of the spheroids.

##### Image Import

The Images module loads and specifies the files to be analyzed by the pipeline. It ensures the selection of desired files within the selected folder. In the Image module, the selection criterion applied was to select only .tiff files, a criterion only necessary if other file types present in the selected folder are not desired for downstream analysis. If it is certain that the selected folder/images are only those of interest, the Filter Images option is set to No-filtering.

In the Metadata module, metadata describing the input images are extracted from the file name. To achieve this, a regular expression (regex) is specified. For example, the file names could be stored in the format Experiment_SpheroidID_Channel.tiff. As such, the regex needed to extract the metadata is in the form (?P<Experiment>.*)_(?P<SpheroidID>.*(_\d+)?)_(?P<Channel>TRANS).tiff. The regex can be modified to suit any different file name.

**Figure 3:**
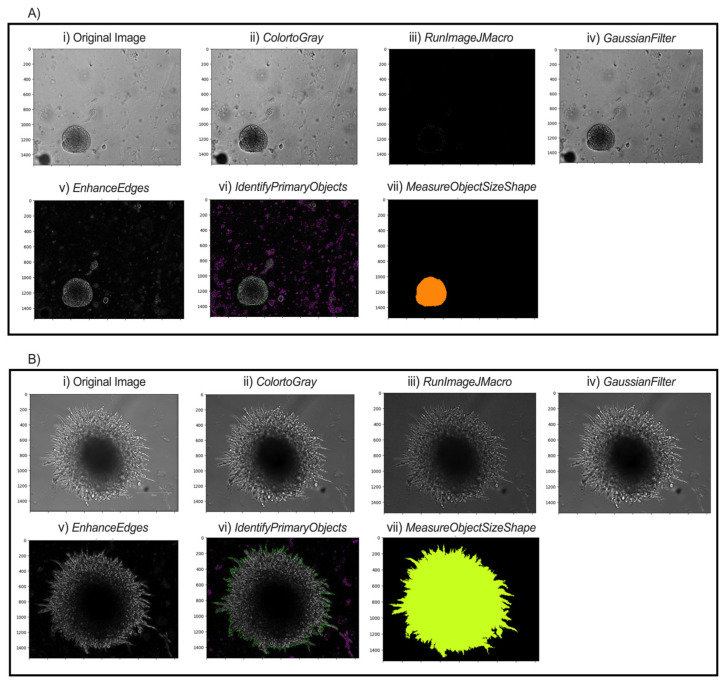
Modular workflow of image analysis pipeline for two possible spheroid morphologies. Representative images of pB2 spheroids (**A**) and pB3 spheroids (**B**) are shown. (**i**) Transverse (TRANS) images of spheroids were captured. (**ii**) All TRANS images were converted into grayscale. (**iii**) Images were sharpened through FIJI to enhance contrast and emphasize object contours. (**iv**) Gaussian smoothing softened the image and reduced high-frequency noise to facilitate thresholding and segmentation. (**v**) Edges of the spheroids were enhanced to allow the software to identify distinct spheroids. (**vi**) Intensity and shape specifications further allowed for the single spheroid to be distinguished from surrounding noise. (**vii**) Final object outline is quantified for data export.

In the NamesAndTypes module, names are assigned to the input images to be referenced by other modules in the pipeline. In this case, TRANS was used (Figure 3A(i),B(i)). The Groups module was not used in this pipeline.

##### Pre-Processing

The ColorToGray module converts all images into grayscale (Figure 3A(ii),B(ii)). Although the images are brightfield images, the ColorToGray module is necessary because it equalizes the relative weights of the channels across all the images. Conversion to grayscale is also necessary because some modules can only process grayscale images. CellProfiler does not inherently have a single module to sharpen the images. As such, RunImageJMacro exports the images from the previous module and executes an ImageJ macro on them. This step requires that ImageJ (FIJI) is already installed on the computer [26]. The directory of the macro and the application (FIJI) should be specified before running the module. Sharpening the image enhances contrast in the image and emphasizes object contours (Figure 3A(iii),B(iii)). GaussianFilter then smooths the image and reduces high-frequency noise, facilitating thresholding and segmentation (Figure 3A(iv),B(iv)). The counterintuitive step of blurring after sharpening helps to smooth out boundaries and reduce noise in the background while keeping object boundaries sharp. A sigma of 1.5–2 is suitable for a good blurring effect. The EnhanceEdges model uses the Sobel method to find and enhance edges of the spheroids. This creates a mask, containing bright objects on a dark background, which allows the software to identify the spheroids distinctly (Figure 3A(v),B(v)).

##### Segmentation

The IdentifyPrimaryObjects module is responsible for identifying objects in the image. Advanced settings allow full customization of the identification methods. Based on the length of spheroid culture and time of initial image capture, the typical minimum and maximum diameters of spheroids are set to 250 and 3000 pixel units, respectively. For images captured during the first few days, objects touching the image border are discarded, since captured and selected spheroids are unlikely to extend beyond the frame. This setting should be adjusted if spheroid images later in the series extend to the borders. Thresholding was performed using the minimum cross-entropy method, which empirically provided the most accurate and consistent segmentation results across our image sets, with threshold smoothing scale and correction factor 0 and 0.7, respectively. The lower and upper bounds on the threshold are 0.02 and 1.0, respectively. The lower bound is small because the inputs for this model are masks from the EnhanceEdges module. When spheroids start to grow out and form “spikes,” the lower bound can be reduced to 0.015 to allow capturing of thinner projections. The method to distinguish clumped objects and the method to draw lines between clumped objects are set as Intensity and Shape, respectively. This combination most accurately distinguishes and identifies objects (Figure 3A(vi),B(vi)).

##### Quantification and Output

Various morphological object features such as area, perimeter, solidity, and major length axis, among several others, are extracted from the object outline (Figure 3A(vii),B(vii)), calculated in the MeasureObjectSizeShape module and reported with the output. In the ExportToSpreadsheet, the destination of the output .csv file is specified. The measurements needed for morphological analysis of spheroid growth characteristics are within the output .csv file.

## 4. Expected Results

### 4.1. Automated Analysis Pipeline to Measure Breast Cancer Spheroids

A hallmark of cancer cells is their ability to undergo invasion and metastasis. Cancer cells can spread from the primary site to distant tissues by transitioning from an epithelial to a mesenchymal morphology, a process known as epithelial–mesenchymal transition (EMT) or epithelial–mesenchymal plasticity (EMP). While the epithelial phenotype is regular and easily defined, the mesenchymal phenotype is mostly irregular, with active construction and deconstruction of actin filaments. In our analysis model, this transition is represented by the change from a compact and uniformly distributed object to a variedly distributed object and the formation of “spikes” along the edges of the object. Quantitatively, the change is shown by measuring the form factor and solidity of the captured spheroids. They are calculated as:

$$Form\;Factor=\frac{4\pi *Area}{{Perimeter}^{2}}$$

$$Solidity=\frac{Object\;Area}{Convex\;Hull\;Area}$$

$$Mesenchymal\;Morphology\;Score=\frac{1}{Form\;Factor*Solidity}$$

The form factor equals 1 for a perfectly circular/round object, while a Form Factor < 1 suggests a more irregular shape. Solidity is a shape descriptor that measures the ratio of the area of an object to the area of its convex hull. It indicates how “full” or compact an object is within its boundary. A higher Solidity value (closer to 1) indicates a more compact object with fewer indentations or concavities, while a lower value suggests a more irregular or porous shape. For each spheroid, we calculate the “mesenchymal morphology score” (MS) using the inverse of the product of the form factor and solidity. The mesenchymal score serves as a morphological proxy for epithelial–mesenchymal transition (EMT) progression. As such, the higher the MS, the more mesenchymal in nature the spheroids appear to be. The results of these measurements, reported in a .csv file, can then be analyzed using Excel or other spreadsheet applications.

To evaluate phenotypic changes in spheroids over time, we applied our image analysis pipeline to obtain the mesenchymal scores of four treatment conditions across multiple time points. For each image, spheroids were automatically segmented, and shape descriptors were measured using the MeasureObjectSizeShape module in CellProfiler. The calculated mesenchymal scores were then averaged per condition and time point.

**Figure 4:**
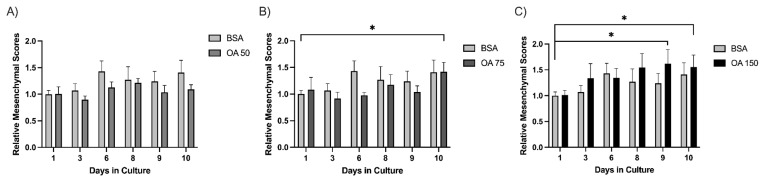
Spheroids exhibit a biphasic response to oleic acid treatment. Mesenchymal scores for spheroids (n = 15) were calculated over 10 days for BSA, OA 50 µM (**A**), OA 75 µM (**B**), and OA 150 µM (**C**) groups. Individual spheroids were tracked over a period of 10 days, and images were processed through the pipeline detailed above. Average mesenchymal scores were normalized to the BSA control on Day day 1. Statistical significance was determined by student’s Student’s *t*-test, * indicates (*p* ≤ 0.05).

Across the experimental days (days 1–10), 15 spheroids were captured and tracked for each treatment (BSA (control), 50 µM, 75 µM, or 150 µM BSA-conjugated oleic acid (OA). We determined the average size of each spheroid for each day using the analysis pipeline. The results demonstrated that 50 µM OA blunts the increase in MS of spheroids relative to BSA (Figure 4A). Interestingly, this suppressive effect is lost on day 10 at 75 µM OA (Figure 4B) and day 3 at 150 µM OA (Figure 4C), indicating a more rapid morphological transition to a mesenchymal phenotype that occurred at higher concentrations of OA. This suggests that administration of OA is suppressive of mesenchymal growth at lower concentrations, but as the concentration of OA increases, the mesenchymal growth of spheroids is enhanced. Thus, by utilizing our 3D cell culture model along with our custom image analysis pipeline, we may have the ability to detect the impact of treatments/therapies on tumor cell behavior in a more physiological three-dimensional context.

### 4.2. Limitations and Future Directions

While we were able to test the hypothesis that unsaturated fat limits the mesenchymal transition in breast cancer cells using our analysis pipeline, we are acutely aware of its limits. One limitation of this approach is its reliance on well-defined/focused spheroid boundaries. Images with poor contrast or overlapping structures may result in inaccurate segmentation. This may be overcome by incorporating fluorescent imaging or further refining segmentation algorithms in CellProfiler. Another limitation of the current workflow is its reliance on complete in-frame imaging (i.e., spheroids must be fully captured within the 10× objective field of view to allow accurate segmentation and analysis). Spheroids that extend beyond the image borders may be incorrectly quantified due to incomplete shape data. This could be addressed by capturing images from the 4× objective view, although finer identification of borders may not be possible. The pipeline also currently requires that users have ImageJ (FIJI) pre-installed on the computer. To potentially streamline the workflow, we plan to integrate native CellProfiler modules to achieve similar functionality. We also note that while a large number of images can be processed through the pipeline at a time, summarizing the output from the pipeline is currently only minimally automated. Data analysis and plotting using MS Excel or other spreadsheet applications is currently done manually.

Future work will involve integrating additional features, such as texture or intensity profiles, into the current pipeline to offer more biological insight. By making use of fluorescent imaging, it is possible to also analyze the growth pattern and morphological characteristics of irregularly shaped spheroids—as well as analyze co-culture models with fluorescence-labeled cells. Additionally, to confirm mesenchymal characteristics, we can use immunostaining for specific mesenchymal markers (N-cadherin, vimentin, and Zeb1) as previously described [23]. To test for the validity of the data generated from the image analysis pipeline, we will also include a protocol to harvest the spheroids after the incubation period to quantify proteins associated with EMT. Incorporating these would offer more biological insight. Our long-term objective with this protocol is to use image-derived morphology scores derived along with robust transcriptomic or proteomic datasets to provide even stronger biological interpretations of tumor cell behavior in 3D culture. Combining that with our bioprinting protocols would give us a medium- to high-throughput method to evaluate the effects of treatments on multiple in vitro 3D tumor models.

## 5. Conclusions

The method described above for generating 3D cultures and combining them with semiautomated analysis is unique because it integrates precise GelMA-based bioprinting with open-source software for easily accessible and reproducible analysis of cancer spheroids. This combination allows controlled spatial deposition of spheroid-laden bioinks, facilitating scalable, physiologically relevant tumor models with robust quantitative morphometric readouts suited for drug screening and tumor microenvironment studies.

Using this method, we found that in 3D culture, PyMT pB2 and pB3 spheroids demonstrate a biphasic response to OA treatment at lower and higher concentrations. Treatment with 50 µM OA prevented an increase in MS compared to BSA (Figure 4A); however, this effect was lost by day 10 at 75 µM OA (Figure 4B) and day 3 at 150 µM OA (Figure 4C). These results indicate lower OA levels may suppress the mesenchymal transition of spheroids, whereas higher OA levels may accelerate the mesenchymal transition of spheroids.

Collectively, our work here demonstrates the utility of the GelMA-based cell culture platform and the image analysis pipeline in bridging the gaps between traditional 2D culture and in vivo systems and contributing to the development of a comprehensive experimental framework that supports the next generation of cancer research.

## Figures and Tables

**Table 1:** Culture medium formulation.

Component Name	Final Concentration	Supplier	Cat. Number
DMEM	Base medium (50%)	Sigma	SIAL-D6429-500ML
F12 nutrient mix	Base medium (50%)	Gibco	11765054
Non-essential amino acids	1×	Cytiva	16777-186
Penicillin–streptomycin	1×	Gibco	15-140-122
Adult bovine serum	5% or 1%	Sigma	B9433-500ML

**Table 2:** Notes for addressing potential issues with droplets.

Problem	Possible Cause	Possible Remedies
There is no flow	Presence of air bubbles in amber cartridge	Remove cartridge from the printer. Insert cartridge piston and push to expel any air bubbles.
	GelMA is too viscous	To reduce viscosity, incubate the cartridge in a horizontal position at 37 °C for 2–4 min.
	Low pressure/ Small extrusion time	Increase pressure/extrusion time setting gradually until desired flow is achieved.
Droplet is too fluid	GelMA is too fluid	Chill the cartridge at 4 °C for 2 min.
	High pressure setting	Reduce pressure until desired consistency is achieved.
	High temperature setting	Reduce temperature by 2–3 °C approaching room temperature, and test to see if desired consistency is achieved.
Low structural integrity of droplets	Low LAP concentration	Increase crosslinking time per droplet or decrease the distance of the light source from the well plate.

## Data Availability

The raw data supporting the conclusions of this article will be made available by the authors on request.

## Abbreviations

The following abbreviations are used in this manuscript.

2D	two-dimensional
3D	three-dimensional
ABS	adult bovine serum
BSA	bovine serum albumin
CO_2_	carbon dioxide
DMEM	Dulbecco’s modified Eagle’s medium
DS	degree of substitution
EDTA	ethylenediaminetetraacetic acid
EMP	epithelial–mesenchymal plasticity
EMT	epithelial–mesenchymal transition
FIJI	Fiji Is Just ImageJ, version 2.14.0
GelMA	gelatin methacryloyl
LAP	lithium phenyl-2,4,6-trimethylbenzoylphosphinate
LED	light-emitting diode
MS	mesenchymal morphology score
OA	oleic acid
OA–BSA	oleic acid–bovine serum albumin conjugate
PBS	phosphate-buffered saline (implied, though not explicitly expanded)
PyMT	polyoma middle T antigen
qM	quasi-mesenchymal
ROI	region of interest
TIFF	Tagged Image File Format
TME	tumor microenvironment
